# Antiteratogenic Effects of **β**-Carotene in Cultured Mouse Embryos Exposed to Nicotine

**DOI:** 10.1155/2013/575287

**Published:** 2013-05-08

**Authors:** Chunmei Lin, Jung-Min Yon, A Young Jung, Jong Geol Lee, Ki Youn Jung, Beom Jun Lee, Young Won Yun, Sang-Yoon Nam

**Affiliations:** College of Veterinary Medicine and Research Institute of Veterinary Medicine, Chungbuk National University, Cheongju 361-763, Republic of Korea

## Abstract

After maternal intake, nicotine crosses the placental barrier and causes severe embryonic disorders and fetal death. In this study, we investigated whether **β**-carotene has a beneficial effect against nicotine-induced teratogenesis in mouse embryos (embryonic day 8.5) cultured for 48 h in a whole embryo culture system. Embryos exposed to nicotine (1 mM) exhibited severe morphological anomalies and apoptotic cell death, as well as increased levels of TNF-**α**, IL-1**β**, and caspase 3 mRNAs, and lipid peroxidation. The levels of cytoplasmic superoxide dismutase (SOD), mitochondrial manganese-dependent SOD, cytosolic glutathione peroxidase (GPx), phospholipid hydroperoxide GPx, hypoxia inducible factor 1**α**, and Bcl-*x*_*L*_ mRNAs decreased, and SOD activity was reduced compared to the control group. However, when **β**-carotene (1 × 10^−7^ or 5 × 10^−7^ *μ*M) was present in cultures of embryos exposed to nicotine, these parameters improved significantly. These findings indicate that **β**-carotene effectively protects against nicotine-induced teratogenesis in mouse embryos through its antioxidative, antiapoptotic, and anti-inflammatory activities.

## 1. Introduction

Cigarette smoking can increase the risk of adverse outcomes during pregnancy, including fetal growth restriction, increased rates of spontaneous abortion, premature placental abruption, perinatal lethality, decreased birth weight, and sudden infant death syndrome [1]. Although the mechanisms linking fetal exposure to cigarette smoke with cellular damage are not clearly understood, smoking induces a toxic state that increased oxidative stress and modulates inflammatory responses [2]. Human and animal studies have demonstrated that cigarette smoking causes oxidative damage and growth retardation in the embryo via production of excess reactive oxygen species (ROS) [3]. Nicotine, a major toxic component of cigarette smoke, crosses the placental barrier and acts directly on the fetus, with fetal concentrations generally 15% higher than maternal levels [1]. 

Maternal cigarette smoking during pregnancy may alter the micronutrient status in both the fetal and maternal environment [4]. Recently, we found that resveratrol, a polyphenol from red wine, can prevent nicotine-induced teratogenesis in cultured mouse fetuses [5]. Dietary micronutrients such as vitamin C, vitamin E, and *β*-carotene contribute to the antioxidant defense system [6]. In particular, the provitamin A carotenoid *β*-carotene is not only an essential source of vitamin A [7], but also shows antioxidative and anti-inflammatory activities in various tissues [8].

Since early embryonic antioxidant systems are immature and antioxidants are present at considerably lower levels than in adults, the developing embryo may be more susceptible to ROS-induced damage [9]. Recently, exogenous antioxidants introduced through diet have become popular. There has been a growing interest in identification of possible dietary antioxidants to treat or prevent diseases caused by ROS. Therefore, we hypothesized that the nicotine-induced excessive ROS that leads to embryonic and fetal oxidative stress could be effectively counteracted by *β*-carotene and oxidative damage could be attenuated. In the present study, we investigated the potential protective effects of *β*-carotene against nicotine-induced teratogenesis in cultured mouse embryos using a whole embryo culture system.

## 2. Materials and Methods

### 2.1. Experimental Animals

Male and female ICR mice (8–10 weeks old) were purchased from a commercial breeder (BioGenomics Co., Seoul, Republic of Korea). The animals were housed in a climate controlled facility with an ambient temperature of 21 ± 2°C, relative humidity of 55 ± 10%, air ventilation rate of 10 cycles per hour, and a 12 : 12 h light : dark cycle. The animals were fed standard mouse chow (Samyang Ltd., Incheon, Republic of Korea) and tap water *ad libitum* throughout the experimental period. One male and three female mice were housed in a cage for mating. Pregnancies were confirmed the following morning (08:00) by the presence of vaginal plugs or spermatozoa detected in a vaginal smear after mating the previous evening (20:00); this was considered embryonic day (E) 0.5. Pregnant mice were sacrificed and embryos were obtained at E8.5. All experiments were approved by the Chungbuk National University Animal Care Committee and carried out according to the Guide for Care and Use of Animals (Chungbuk National University Animal Care Committee, according to NIH number 86–23). 

### 2.2. Rat Serum Preparation

Serum of Sprague-Dawley male rats (10–12 weeks old) was prepared for embryo cultures as follows. After collection, blood samples were immediately centrifuged for 10 min at 3,000 rpm and 4°C to clear the plasma fraction of cells. The supernatant was then transferred to new tubes and centrifuged for 10 min at 3,000 rpm and 4°C to remove remaining blood cells. The clear serum supernatant was decanted and pooled, and the pooled serum was heat-inactivated for 30 min at 56°C in a water bath. It was then either used immediately or stored at −70°C. Serum was incubated at 37°C and filtered through a 0.2 *μ*m filter prior to use in the whole embryo culture.

### 2.3. Whole Embryo Culture and Nicotine and *β*-Carotene Treatments

The whole embryo culture system was based on a previously described procedure [10]. Animals were sacrificed between 09:00 and 10:00 h via cervical dislocation when embryos reached E8.5. Only embryos with 4–8 somites were utilized. After removing the decidua and Reichert's membranes, embryos with intact visceral yolk sacs and ectoplacental cones were placed randomly into sealed culture bottles (three embryos/bottle) containing 3 mL of culture medium and different concentrations (1 × 10^−7^ or 5 × 10^−7^ *μ*M) of *β*-carotene (Sigma, St. Louis, MO, USA) dissolved in dimethyl sulfoxide (DMSO, Sigma) and/or 1 mM nicotine (163.8 *μ*g/mL serum; Sigma). The nicotine concentration used here was determined by previous studies [5, 11]. The final concentration of DMSO in the medium was less than 0.1%. Embryos were randomized into four treatment groups: (1) control, (2) nicotine, (3) nicotine plus 1 × 10^−7^ *μ*M *β*-carotene, and (4) nicotine plus 5 × 10^−7^ *μ*M *β*-carotene. The embryos were incubated at 37 ± 0.5°C in sealed culture bottles (three embryos/bottle) and rotated at 25 rpm. The culture bottles were initially gassed with a mixture of 5% O_2_, 5% CO_2_, and 90% N_2_ over a 17 h period at a flow rate of 150 mL/min. Subsequent gassing was performed at the same rate over 7 h (20% O_2_, 5% CO_2_, and 75% N_2_) and 24 h (40% O_2_, 5% CO_2_, and 55% N_2_). All embryos were cultured for 48 h using a whole embryo culture system (Ikemoto Rika Kogyo, Japan). 

### 2.4. Morphological Scoring

At the end of the 48 h culture period, the morphology of the embryos was evaluated according to a previously described scoring system [12]. Only viable embryos with yolk sac circulation and a heartbeat were used for morphological scoring. Measurements of each viable embryo were obtained for 17 standard scoring items, as well as the yolk sac diameter, crown-rump length, and head length. The morphological features that were assessed included embryonic flexion, heart, caudal neural tube, brain (forebrain, midbrain, and hindbrain), otic and optic systems, olfactory organs, branchial arch, maxilla, mandible, limb buds (forelimb and hindlimb buds), yolk sac circulation, allantois, and somites. 

### 2.5. Lipid Peroxidation Measurements

Lipid peroxidation was determined using thiobarbituric acid (TBA) as described by Ohkawa et al. [13] with minor modifications. The level of malondialdehyde (MDA), a secondary by-product of lipid peroxidation, was measured spectrophotometrically after reaction with TBA. The results are expressed as nmol/mg protein. Briefly, embryos (10–16) in each group were homogenized in chilled 10 mM phosphate buffer and were then mixed thoroughly with 8.1% sodium dodecyl sulfate, 20% acetic acid, and 0.75% 2-thiobarbituric-acid solution. The solution was heated for 30 min in a 95°C oven. After cooling, insoluble material was removed by centrifugation at 3500 rpm for 15 min. The absorbance of the supernatant was measured at 532 nm with a spectrophotometer and compared to the prepared 1,1,3,3,-tetramethoxypropane standard curve. The total protein content of the embryos was determined according to the method of Lowry et al. [14] using bovine serum albumin as the standard.

### 2.6. SOD Activity Assay

Total SOD activity was assayed using a SOD Assay kit-WST (Dojindo Laboratories, Kumamoto, Japan). Briefly, 5–8 mouse embryos were homogenized, and the protein concentrations of the supernatants were analyzed by the Bradford method [15]. The supernatants were incubated with an assay reagent containing xanthine, xanthine oxidase, and a water-soluble tetrazolium salt, WST-1. The superoxide free radicals generated from the xanthine by xanthine oxidase reduced WST-1 to WST-1 diformazan, which absorbs maximally at 450 nm. SOD in the embryos inhibits the WST-1 reduction, since the enzyme catalyzes the dismutation of superoxide ions to molecular oxygen and hydrogen peroxide. The reduction of WST-1 was measured spectrophotometrically at 450 nm. SOD activity was calculated as an inhibition rate in which 1 U was defined as a 50% decrease from the control value over a period of 30 min at 37°C. 

### 2.7. Nile Blue Staining

Embryonic cell death was detected by a classic technique using Nile blue staining to observe apoptotic nuclei and dead cells in blue color. E10.5 embryos were dissected into PBS. Embryos were placed in 1.5% Nile blue (Sigma) in PBS, incubated at 37°C for 45 minutes, and then monitored every 15 min using a light microscope until staining reached the desired level. Pale blue staining (background level) shows the normal live cells and dark blue staining reveals regions of cell death.

### 2.8. Quantitative Real-Time Polymerase Chain Reaction (PCR) Analysis

Total RNA was extracted from six to nine cultured mouse embryos using the Trizol Reagent (Invitrogen, Carlsbad, CA, USA). The RNA was further purified using an RNA clean-up kit (Macherey-Nagel, Bethlehem, USA). Total RNA (2 *μ*g) was used for cDNA synthesis (Invitrogen). Real-time PCR was carried out in a 20 *μ*L reaction volume using the SYBR Green Master Mix (Applied Biosystems, Foster City, CA, USA) and mouse embryonic cDNA (1.6 *μ*g) as the template. Reactions were performed using a 7500 Real-Time PCR System (Applied Biosystems), according to the manufacturer's instructions. Gene-specific primers were designed by TIB Mol-Bio Synthesis (Berlin, Germany). Primers to mouse cytoplasmic superoxide dismutase (SOD1), manganese SOD (SOD2), cytoplasmic glutathione peroxidase (GPx1), phospholipid hydroperoxide glutathione peroxidase (GPx4), hypoxia inducible factor 1*α* (HIF-1*α*), Bcl-*x*_*L*_, caspase 3, and cytokines (TNF-*α* and IL-1*β*) were used (Table 1). *β*-actin primers were used as an internal standard to normalize target transcript expression. Data from nine independent runs were analyzed using the comparative Ct method [16].

### 2.9. Statistical Evaluation

Group differences in gene expression, lipid peroxidation, and SOD activity were assessed via one-way ANOVA followed by Tukey's multiple comparison test. Morphological data were compared using the Kruskal-Wallis nonparametric ANOVA and Dunn's multiple comparison *post hoc* test. A *P* < 0.05 was considered significant. All data are expressed as mean ± SEM. All analyses were conducted using the SPSS for Windows software, version 10.0 (SPSS Inc., Chicago, IL, USA).

## 3. Results

### 3.1. Effect of *β*-Carotene on Nicotine-Induced Developmental Arrest in Mouse Embryos

**Figure 1:**
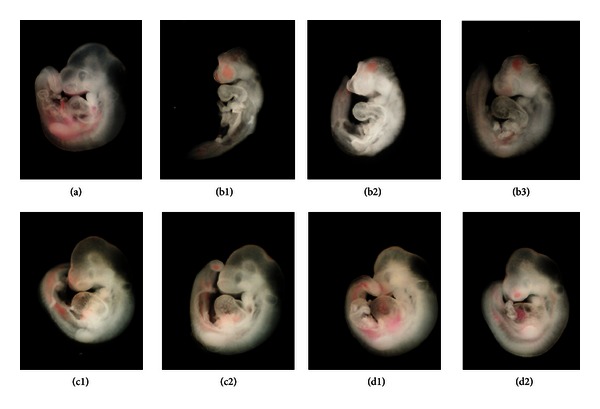
Representative images of mouse embryos exposed to nicotine and *β*-carotene. Normal control group (a). Embryos treated with 1 mM nicotine alone show typical abnormalities such as exposed brain, reduced forebrain, abnormal heart, deformed posterior trunk, and regressed forelimbs (b1−3). Embryos treated with nicotine plus *β*-carotene [1 × 10^−7^ *μ*M (c1 and c2) and 5 × 10^−7^ *μ*M (d1 and d2)] appear morphologically similar to the control group.

Growth parameters, including yolk sac diameter and circulation, size of the allantois, crown-rump length, head length, and number of somites, and developmental parameters, including morphology of the heart, hind-, mid-, and forebrain, otic, optic, and olfactory systems, branchial bars, maxillary and mandibular processes, forelimb, and hindlimb, of mouse embryos exposed to nicotine in the presence or absence of *β*-carotene were scored according to an established scale [12] (Table 2 and Figure 1). All the growth and developmental parameters of the nicotine-treated group were significantly lower than the normal controls (*P* < 0.05). Furthermore, the total morphological score (48.4 ± 0.81) of embryos exposed to nicotine alone was significantly lower than that of control embryos (75.0 ± 0.46; *P* < 0.05). However, when *β*-carotene (1 × 10^−7^ or 5 × 10^−7^ *μ*M) was added to the culture medium in the presence of nicotine (1 mM), the embryos showed significant improvement in all embryonic growth and developmental parameters (*P* < 0.05 compared to nicotine alone), with the exception of the caudal neural tube score. Furthermore, the total morphological score (61.6 ± 0.54 or 62.4 ± 0.72) for each concentration of *β*-carotene was significantly higher than the score for embryos treated with nicotine alone (*P* < 0.05). 

### 3.2. Effect of *β*-Carotene on Nicotine-Induced Oxidative Damage in Mouse Embryos

**Figure 2:**
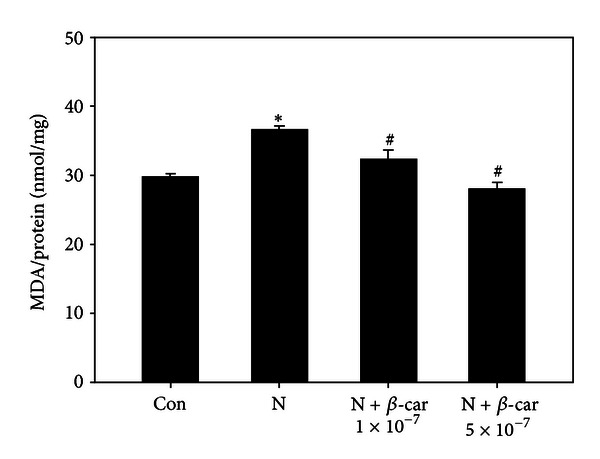
Protective effects of *β*-carotene against oxidative damage induced by nicotine in E8.5 mouse embryos treated *in vitro* for 2 days. Lipid peroxidation was evaluated by measuring the malondialdehyde (MDA) concentration in embryos treated with 1 mM nicotine in the absence or presence of 1 × 10^−7^ or 5 × 10^−7^ *μ*M *β*-carotene (*β*-car). Results are presented as mean ± SEM (*n* = 12). Significant differences (*control versus nicotine alone; ^#^nicotine versus *β*-car + nicotine) were evaluated by one-way ANOVA at *P* < 0.05.

Oxidative stress was analyzed in whole embryos by measuring the MDA levels (Figure 2). Mouse embryos exposed to 1 mM nicotine alone exhibited significantly increased lipid peroxidation (36.63 ± 0.57 nmol/mg) compared to the control group (29.81 ± 0.48 nmol/mg) (*P* < 0.05). However, embryos treated with nicotine plus *β*-carotene (1 × 10^−7^ or 5 × 10^−7^ *μ*M) exhibited significantly reduced lipid peroxidation levels (32.36 ± 1.34 or 28.01 ± 0.96 nmol/mg) compared to the nicotine only group (*P* < 0.05).

### 3.3. *β*-Carotene Enhances SOD Activity in Mouse Embryos Treated with Nicotine

**Figure 3:**
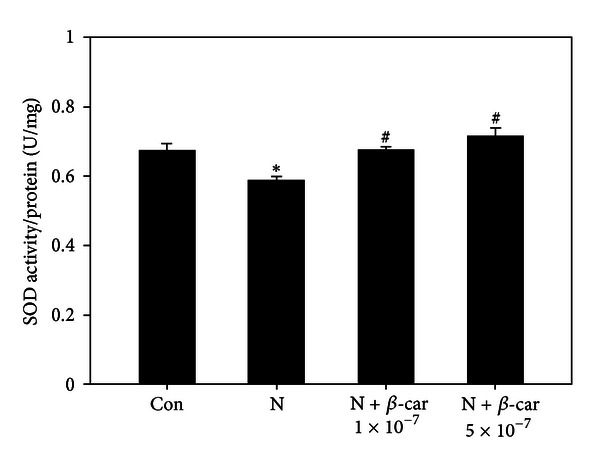
Superoxide dismutase (SOD) activity levels in E8.5 mouse embryos exposed to nicotine and *β*-carotene for 2 days *in vitro*. SOD activity in embryos treated with 1 mM nicotine in the absence or presence of 1 × 10^−7^ or 5 × 10^−7^ *μ*M *β*-carotene (*β*-car) was measured. Results are presented as mean ± SEM (*n* = 6). Significant differences (*control versus nicotine alone; ^#^nicotine versus *β*-car + nicotine) were evaluated by one-way ANOVA at *P* < 0.05.

Mouse embryos exposed to 1 mM nicotine exhibited significantly reduced SOD activity (0.58 ± 0.03 U/mg protein) compared to the control group (0.68 ± 0.04 U/mg protein) (*P* < 0.05). However, when the embryos were treated with 1 × 10^−7^ or 5 × 10^−7^ *μ*M *β*-carotene in the presence of nicotine, SOD activity (0.67 ± 0.02 U/mg or 0.71 ± 0.05 U/mg) was significantly greater than in the nicotine only treatment group (*P* < 0.05) (Figure 3).

### 3.4. *β*-Carotene Upregulates the Expression of Antioxidative Enzyme Genes in Mouse Embryos Exposed to Nicotine

**Figure 4:**
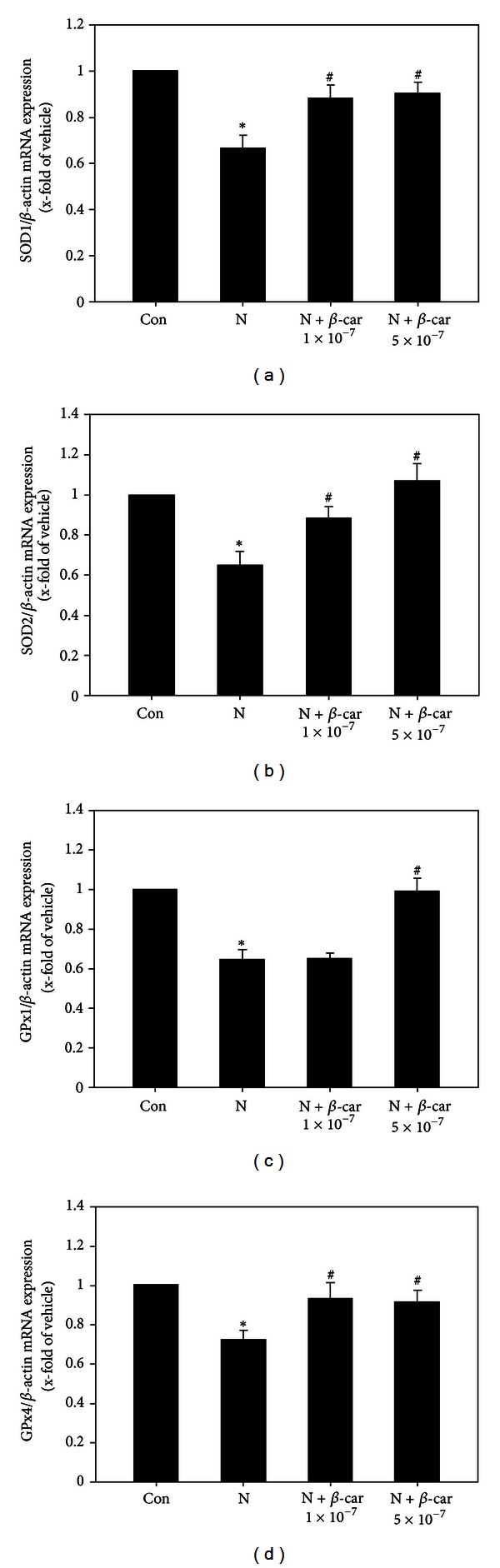
Gene expression levels of antioxidant enzymes in E8.5 mouse embryos exposed to nicotine and *β*-carotene for 2 days *in vitro*. Levels of mRNA for cytoplasmic superoxide dismutase (SOD1, (a)), manganese SOD (SOD2, (b)), cytoplasmic glutathione peroxidase (GPx1, (c)), and phospholipid hydroperoxide GPx (GPx4, (d)) in embryos exposed to 1 mM nicotine in the absence or presence of 1 × 10^−7^ or 5 × 10^−7^ *μ*M *β*-carotene (*β*-car) were measured by quantitative RT-PCR. Results are mean ± SEM (*n* = 8). *β*-actin was used as an internal standard to normalize target transcript expression. Significant differences (*control versus nicotine alone; ^#^nicotine versus *β*-car + nicotine) were evaluated by one-way ANOVA at *P* < 0.05.

The cytoplasmic SOD1 mRNA level (Figure 4(a)) in mouse embryos exposed to 1 mM nicotine was 0.66-fold that of the control group (1-fold). However, when embryos were treated with 1 × 10^−7^ or 5 × 10^−7^ *μ*M *β*-carotene and 1 mM nicotine, the embryo SOD1 mRNA levels (0.89-fold or 0.90-fold that of controls, resp.) were significantly greater than with the nicotine only treatment (*P* < 0.05).

The mitochondrial SOD2 mRNA level (Figure 4(b)) in mouse embryos exposed to 1 mM nicotine was 0.65-fold that of the control group (1-fold). However, when embryos were treated with 1 × 10^−7^ or 5 × 10^−7^ *μ*M *β*-carotene and 1 mM nicotine, the SOD2 mRNA levels (0.88-fold or 1.07-fold that of the control group, resp.) were significantly greater than with the nicotine treatment alone (*P* < 0.05).

The cytoplasmic GPx1 mRNA level (Figure 4(c)) in mouse embryos exposed to 1 mM nicotine was 0.65-fold that of the control group (1-fold) (*P* < 0.05). However, when embryos were treated with 5 × 10^−7^ *μ*M *β*-carotene and 1 mM nicotine, the GPx1 mRNA level (0.99-fold that of the control group) was significantly greater than with the nicotine treatment alone (*P* < 0.05).

The phospholipid hydroperoxide GPx4 mRNA level (Figure 4(d)) in mouse embryos exposed to 1 mM nicotine decreased significantly to 0.72-fold that of the control group (1-fold) (*P* < 0.05). However, when embryos were treated with 1 × 10^−7^ or 5 × 10^−7^ *μ*M *β*-carotene and 1 mM nicotine, the GPx4 mRNA levels (0.93-fold or 0.91-fold that of the control group, resp.) were significantly greater than with the nicotine treatment alone (*P* < 0.05).

### 3.5. *β*-Carotene Upregulates HIF-1*α* Gene Expression in Nicotine-Treated Embryos

**Figure 5:**
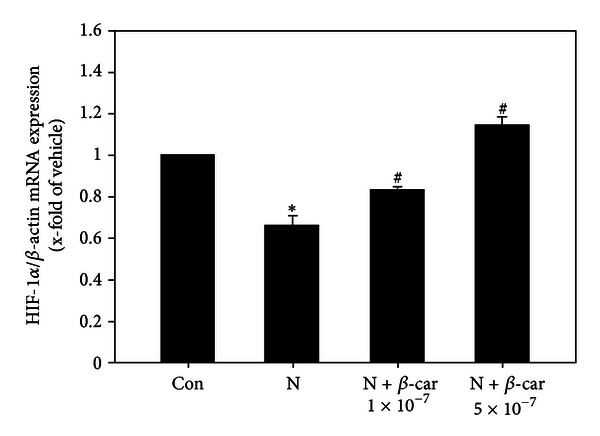
Hypoxia inducible factor-1 *α* expression levels in E8.5 mouse embryos exposed to nicotine and *β*-carotene for 2 days *in vitro*. HIF-1*α* mRNA in embryos exposed to 1 mM nicotine in the absence or presence of 1 × 10^−7^ or 5 × 10^−7^ *μ*M *β*-carotene (*β*-car) was measured by quantitative RT-PCR. Results are mean ± SEM (*n* = 8). *β*-actin was used as an internal standard to normalize target transcript expression. Significant differences (*control versus nicotine alone; ^#^nicotine versus *β*-car + nicotine) were evaluated by one-way ANOVA at *P* < 0.05.

The HIF-1*α* mRNA level in mouse embryos exposed to 1 mM nicotine decreased significantly to 0.66-fold that of the control group (1-fold) (*P* < 0.05). However, when embryos were treated with 1 × 10^−7^ or 5 × 10^−7^ *μ*M *β*-carotene and 1 mM nicotine, HIF-1*α* mRNA levels (0.82-fold or 1.14-fold that of the control group, resp.) were significantly greater than with the nicotine treatment alone (*P* < 0.05) (Figure 5).

### 3.6. *β*-Carotene Downregulates Proinflammatory Cytokines Gene Expression in Embryos Exposed to Nicotine

**Figure 6:**
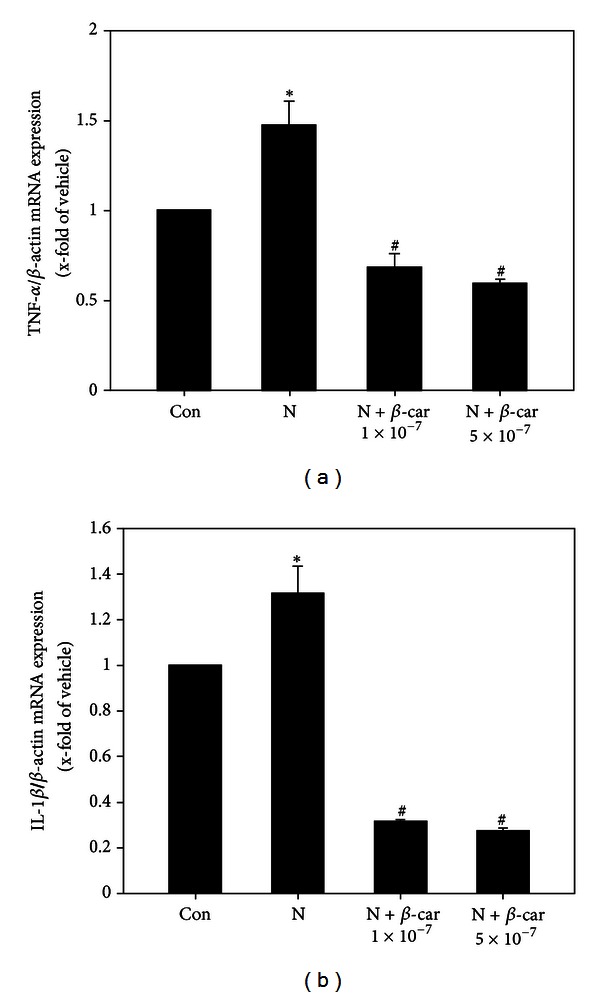
Gene expression levels of proinflammatory cytokines in E8.5 mouse embryos exposed to nicotine and *β*-carotene for 2 days *in vitro*. Levels of TNF-*α* (a) and IL-1*β* (b) mRNA in embryos exposed to 1 mM nicotine in the absence or presence of 1 × 10^−7^ or 5 × 10^−7^ *μ*M *β*-carotene (*β*-car) were measured by quantitative RT-PCR. Results are mean ± SEM (*n* = 8). *β*-actin was used as an internal standard to normalize target transcript expression. Significant differences (*control versus nicotine alone; ^#^nicotine versus *β*-car + nicotine) were evaluated by one-way ANOVA at *P* < 0.05.

The TNF-*α* mRNA level (Figure 6(a)) in mouse embryos exposed to 1 mM nicotine was 1.47-fold that of the control group (1-fold) (*P* < 0.05). However, when embryos were treated with 1 × 10^−7^ or 5 × 10^−7^ *μ*M *β*-carotene and 1 mM nicotine, TNF-*α* mRNA levels (0.68-fold or 0.59-fold that of the control group, resp.) were significantly lower than with the nicotine only treatment (*P* < 0.05).

The IL-1*β* mRNA level (Figure 6(b)) in mouse embryos exposed to 1 mM nicotine was 1.31-fold that of the control group (1-fold) (*P* < 0.05). However, when embryos were treated with 1 mM nicotine and 1 × 10^−7^ or 5 × 10^−7^ *μ*M *β*-carotene, the IL-1*β* mRNA level (0.32-fold or 0.27-fold that of the control group, resp.) was significantly lower than with the nicotine only treatment (*P* < 0.05).

### 3.7. *β*-Carotene Decreases Nicotine-Induced Apoptosis

#### 3.7.1. Bcl-*x*_*L*_ Gene Expression Pattern

**Figure 7:**
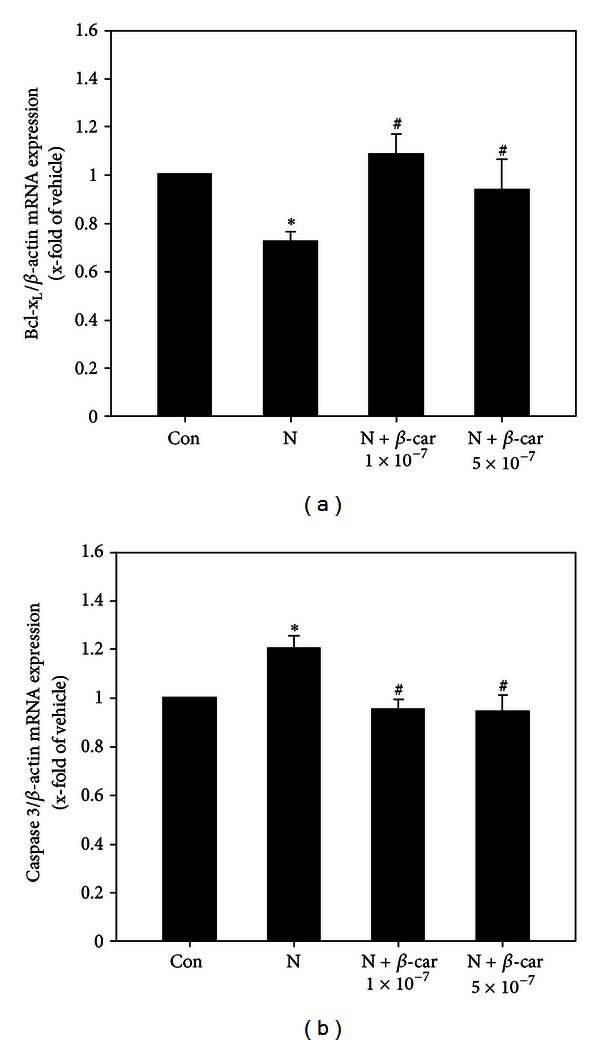
Gene expression levels of apoptosis related factors in E8.5 mouse embryos exposed to nicotine and *β*-carotene for 2 days *in vitro*. Levels of Bcl-*x*_*L*_ (a) and caspase 3 (b) mRNA in embryos exposed to 1 mM nicotine in the absence or presence of 1 × 10^−7^ or 5 × 10^−7^ *μ*M *β*-carotene (*β*-car) were measured by quantitative RT-PCR. Results are mean ± SEM (*n* = 8). *β*-actin was used as an internal standard to normalize target transcript expression. Significant differences (*control versus nicotine alone; ^#^nicotine versus *β*-car + nicotine) were evaluated by one-way ANOVA at *P* < 0.05.

The Bcl-*x*_*L*_ mRNA level in mouse embryos exposed to 1 mM nicotine was 0.72-fold that of the control group value (1-fold) (*P* < 0.05). However, when embryos were treated with 1 mM nicotine and 1 × 10^−7^ or 5 × 10^−7^ *μ*M *β*-carotene, the Bcl-*x*_*L*_ mRNA level (1.09-fold or 0.94-fold that of the control group, resp.) was significantly greater than with the nicotine only treatment (*P* < 0.05, Figure 7(a)).

#### 3.7.2. Caspase 3 Gene Expression Pattern

The caspase 3 mRNA level in mouse embryos exposed to 1 mM nicotine was 1.20-fold that of the control group (1-fold) (*P* < 0.05). However, when embryos were treated with nicotine in the presence of 1 × 10^−7^ or 5 × 10^−7^ *μ*M *β*-carotene, caspase 3 mRNA levels (0.96-fold or 0.94-fold that of the control group, resp.) were significantly lower than with the nicotine only treatment (*P* < 0.05, Figure 7(b)).

#### 3.7.3. *β*-Carotene Reduces Nicotine-Induced Apoptosis in Mouse Embryos

**Figure 8:**
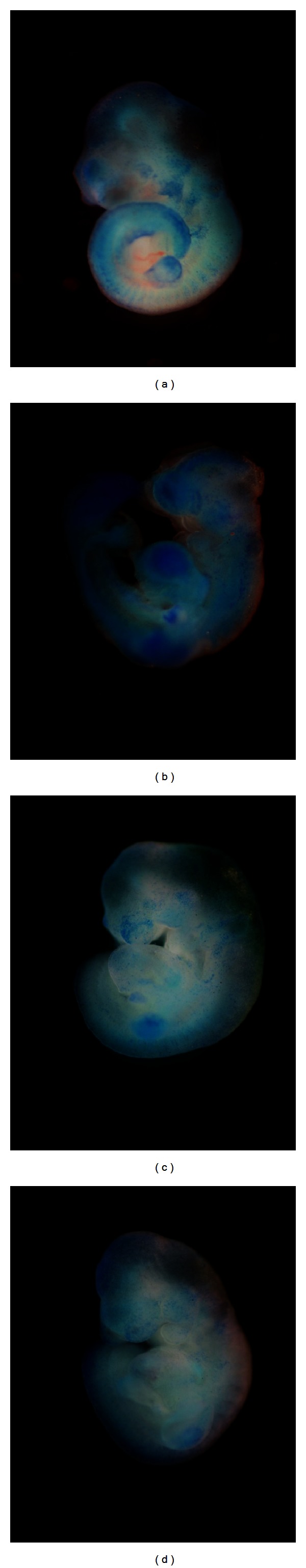
Representative images of apoptotic embryos exposed to nicotine and *β*-carotene by Nile blue staining. Nile blue staining was performed to observe apoptotic nuclei and dead cells which stained dark blue. Normal control embryos (a). Embryos treated with 1 mM nicotine exhibit increased levels of apoptosis (b). Embryos treated with 1 mM nicotine plus *β*-carotene [1 × 10^−7^ *μ*M (c) and 5 × 10^−7^ *μ*M (d)] appear similar to the control group.

To determine whether *β*-carotene antagonizes nicotine-induced apoptosis, the Nile blue staining technique was used. Normal cells were stained pale blue in control embryos (Figure 8(a)). By contrast, apoptotic cells appeared dark blue in color, especially in the heart, optic and olfactory pits, brain, otic stalk, cranial nerve nuclei, and tail bud in the nicotine-treated embryos (Figure 8(b)). Cotreatment with *β*-carotene resulted in a marked reduction in the levels of apoptosis induced by nicotine (Figures 8(c) and 8(d)). 

## 4. Discussion

The popularity of smoking during pregnancy is between 13% and 25% in high-income countries, and is increasing rapidly in low- and middle-income countries [17]. Although effective smoking cessation strategies during pregnancy are important for maternal and fetal health, previous studies have suggested that an alternative therapy may be to use natural antioxidant treatments that can protect against nicotine-induced embryo toxicity [5, 18]. In the present study, we expanded upon this concept not only to demonstrate the beneficial effects of *β*-carotene against nicotine-induced damage, but also to distinguish the mechanisms of nicotine damage further using an embryo culture system.

In previous studies, maternal smoking affected the development of many fetal organs and tissues including the nervous, cardiovascular, and skeletal systems [19–24]. Nicotine increases the fetal heart rate, reduces fetal breathing movements, and is associated with deficiencies in brain cell number [25]. In the current study, embryonic growth, as measured by yolk sac diameter and circulation, size of the allantois, crown-rump length, head length, and number of somites, as well as development of the heart, central nervous system, sensory organs, branchial bars, maxillary and mandibular processes, and limbs were inhibited and morphological features of the embryos were significantly altered by nicotine treatment. However, when nicotine-treated embryos were concurrently exposed to *β*-carotene, most of the morphological anomalies, including abnormal heart development, deformed posterior trunk, regressed limbs, and brain malformations were significantly improved compared to embryos treated with nicotine alone. These findings indicate that *β*-carotene can effectively protect embryos from nicotine-induced defects in organogenesis.

Cell membranes contain substantial levels of polyunsaturated fatty acids that are highly vulnerable to peroxidative breakdown [26]. Oxidative stress characterized by increased ROS and impaired antioxidant defenses acts as an important mediator of defective embryo development and growth retardation [9]. However, both enzymatic (SOD, GPx, and catalase) and nonenzymatic (GSH/GSSG, peroxiredoxin, thioredoxin, vitamin C, and vitamin E) antioxidant systems exist to combat excessive ROS generation [27]. Nicotine induces oxidative stress both *in vivo* and *in vitro* [9]. Recently, we found that resveratrol, a natural polyphenol compound, prevents nicotine-induced teratogenesis in cultured mouse embryos through its potent antioxidative activity [5]. In the current study, nicotine increased the MDA level and decreased the SOD activity in embryos. However, when the embryos were concurrently treated with nicotine and *β*-carotene, these embryonic oxidative stress responses and impaired antioxidant enzyme levels recovered to the control levels. The antioxidant *β*-carotene provides essential protection against oxygen radical damage, since it terminates peroxidative chain reactions of unsaturated lipids in the brain and other tissues [28] and effectively scavenges ROS in cells exposed to oxidative stress [29]. Therefore, exogenous *β*-carotene may improve the SOD status of embryos and neutralize the excess ROS generated by nicotine. 

SODs inactivate superoxide radicals and GPxs reduce hydrogen peroxide to H_2_O at the expense of glutathione oxidation [30, 31]. During mouse embryogenesis, antioxidant enzymes such as GPx1, GPx4, SOD1, and SOD2 are highly expressed in metabolically active tissues [32–35]. In the current study, nicotine significantly decreased SOD1, SOD2, GPx1, and GPx4 gene expression in cultured embryos, but the expression levels were restored by cotreatment with *β*-carotene. As early organogenesis occurs in a relatively hypoxic environment, embryos are sensitive to oxidative stress [9]. Null mutations in HIF-1*α* cause cardiac, vascular, and neural malformations and result in fetal lethality on E10.5 [36]. Hypoxia induces oxidative stress and abnormal organogenesis in mouse embryos by downregulating HIF-1*α* and intracellular SOD gene expression [37]. In the current study, the levels of HIF-1*α* mRNA in cultured embryos decreased significantly following nicotine treatment, but were restored by co-treatment with *β*-carotene. These results indicate that *β*-carotene can protect embryos against nicotine-induced oxidative damage through its antioxidative and antihypoxic activities.

Cigarette smoke alters a wide range of immunological functions and adversely influences humoral and cellular immune responses in both humans and animals [38]. ROS mediate these immune reactions through various proinflammatory cytokines and can influence the function of oocyte, sperm, and embryo [39]. In the current study, nicotine significantly increased gene expression of the proinflammatory cytokines TNF-*α* and IL-1*β* in cultured embryos, but these levels were significantly reduced, to levels lower than control levels, by co-treatment with *β*-carotene. These results indicate that *β*-carotene may protect the embryos by reducing the immune response stimulated by nicotine treatment.

Previous studies have confirmed that apoptosis plays an important role in normal embryonic development. Developmental apoptosis is a well-balanced process that is crucial for formation of embryonic structures. However, interference with this balance induces morphological abnormalities [40, 41]. In the current study, Bcl-*x*_*L*_, one of several antiapoptotic proteins that are members of the Bcl-2 family of proteins, decreased significantly and caspase 3, a marker for cells undergoing apoptosis [42], increased significantly following nicotine treatment of cultured embryos. Increased apoptosis was also detected in embryos exposed to nicotine by Nile blue staining. However, these apoptotic changes induced by nicotine were blocked by co-treatment with *β*-carotene. These results indicate that *β*-carotene protects embryos from nicotine-induced abnormal development via its antiapoptotic activity.

## 5. Conclusions

Nicotine induces excessive ROS and leads to fetal anomalies and lethality. The findings of the current study indicate that the antioxidative, anti-hypoxic, antiapoptotic, and antiproinflammatory functions of *β*-carotene may prevent nicotine-induced impairments of embryos and facilitate normal embryonic development. Although these data support the hypothesis that *β*-carotene obtained in the diet effectively counteracts the deleterious effects of nicotine during fetal organogenesis, an *in vivo* study using mouse would be needed to compare the functions of *β*-carotene on nicotine-induced embryotoxicities in future. 

## Figures and Tables

**Table 1:** Primer sequences used in the study.

Gene	Primer sequence (5′-3′)	Accession number
*β*-actin	Forward: TTT CCA GCC TTC CTT CTT GGG TAT G	NM_007393
	Reverse: CAC TGT GTT GGC ATA GAG GTC TTA C	
SOD1	Forward: TGC GTG CTG AAG GGC GAC	NM_011434
	Reverse: GTC CTG ACA ACA CAA CCT GGT TC	
SOD2	Forward: GGA GCA AGG TCG CTT ACA GA	NM_013671
	Reverse: GTG CTC CCA CAC GTC AAT C	
GPx1	Forward: TGT TTG AGA AGT GCG AAG TG	NM_008160
	Reverse: GTG TTG GCA AGG CAT TCC	
GPx4	Forward: TAA GAA CGG CTG CGT GGT	NM_008162
	Reverse: GTA GGG GCA CAC ACT TGT AGG	
HIF-1*α*	Forward: CAC CAG ACA GAG CAG GAA	NM_010431
	Reverse: TCA GGA ACA GTA TTT CTT TGA TTC A	
TNF-*α*	Forward: TACCTTGTTGCCTCCTCTT	NM_013693
	Reverse: GTCACCAAATCAGCGTTATTAAG	
IL-1*β*	Forward: TCACAAGCAGAGCACAAG	NM_008361
	Reverse: GAAACAGTCCAGCCCATAC	
Bcl-*x*_*L*_	Forward: TGACCACCTAGAGCCTTGGA	NM_009743
	Reverse: TGTTCCCGTAGAGATCCACAA	
Caspase 3	Forward: AAA GCC GAA ACT CTT CA TCA T	NM_009810
	Reverse: GTC CCA CTG TCT GTC TCA	

**Table 2:** Summary of morphological changes in cultured mouse embryos exposed to 1 mM nicotine in the presence or absence of 1 × 10^−7^ or 5 × 10^−7^ *µ*M *β*-carotene (*β*-car).

Chemical (dose)	Con	N	N + *β*-car (1 × 10^−7^)	N + *β*-car (5 × 10^−7^)
Number of embryos	33	33	34	31

Yolk sac diameter (mm)	3.5 ± 0.30	2.4 ± 0.29^a^	3.0 ± 0.32^b^	2.9 ± 0.21^b^
Yolk sac circulation	4.3 ± 0.37	3.6 ± 0.64^a^	4.0 ± 0.26	3.9 ± 0.23^b^
Allantois	2.3 ± 0.44	1.6 ± 0.26^a^	1.9 ± 0.19^b^	2.0 ± 0.15^b^
Flexion	4.9 ± 0.12	3.2 ± 1.00^a^	4.7 ± 0.76^b^	4.9 ± 0.19^b^
Crown-rump length (mm)	3.0 ± 0.29	2.1 ± 0.29^a^	2.6 ± 0.22^b^	2.4 ± 0.25^b^
Head length (mm)	1.5 ± 0.21	0.9 ± 0.18^a^	1.3 ± 0.17^b^	1.2 ± 0.14^b^
Heart	4.8 ± 0.33	3.4 ± 0.55^a^	4.5 ± 0.36^b^	4.3 ± 0.35^b^
Hindbrain	4.7 ± 0.29	2.9 ± 0.60^a^	4.2 ± 0.29^b^	4.2 ± 0.20^b^
Midbrain	4.9 ± 0.19	2.9 ± 0.57^a^	4.2 ± 0.23^b^	4.2 ± 0.24^b^
Forebrain	5.8 ± 0.33	3.0 ± 0.56^a^	4.4 ± 0.33^b^	4.3 ± 0.36^b^
Otic system	4.9 ± 0.17	3.0 ± 0.58^a^	4.3 ± 0.25^b^	4.4 ± 0.33^b^
Optic system	5.0 ± 0.09	2.9 ± 0.57^a^	4.3 ± 0.24^b^	4.4 ± 0.30^b^
Branchial bars	3.7 ± 0.31	2.2 ± 0.38^a^	3.1 ± 0.36^b^	3.2 ± 0.38^b^
Maxillary process	2.9 ± 0.25	1.3 ± 0.41^a^	2.1 ± 0.25^b^	2.2 ± 0.45^b^
Mandibular process	2.8 ± 0.34	1.3 ± 0.39^a^	2.0 ± 0.30^b^	2.1 ± 0.39^b^
Olfactory system	2.8 ± 0.30	0.4 ± 0.50^a^	1.8 ± 0.40^b^	1.7 ± 0.56^b^
Caudal neural tube	5.0 ± 0.00	4.6 ± 0.72	5.0 ± 0.00	5.0 ± 0.00^b^
Fore limb	2.8 ± 0.21	1.7 ± 0.45^a^	2.6 ± 0.39^b^	2.7 ± 0.39^b^
Hind limb	1.2 ± 0.63	0.0 ± 0.00^a^	0.6 ± 0.45^b^	0.7 ± 0.52^b^
Somites	4.0 ± 0.00	3.5 ± 0.51^a^	4.0 ± 0.00^b^	4.0 ± 0.00^b^

Total score	75.0 ± 0.46	48.4 ± 0.81^a^	61.6 ± 0.54^b^	62.4 ± 0.72^b^

Each value represents the mean ± SEM.

^a^Versus normal control (Con) group at *P*< 0.05.

^b^Versus nicotine alone (N) group at *P*< 0.05.
